# A Family of Indoles Regulate Virulence and Shiga Toxin Production in Pathogenic *E. coli*

**DOI:** 10.1371/journal.pone.0054456

**Published:** 2013-01-23

**Authors:** Bettina Bommarius, Akwasi Anyanful, Yevgeniy Izrayelit, Shantanu Bhatt, Emily Cartwright, Wei Wang, Alyson I. Swimm, Guy M. Benian, Frank C. Schroeder, Daniel Kalman

**Affiliations:** 1 Department of Laboratory Medicine and Pathology, Emory University, Atlanta, Georgia, United States of America; 2 Boyce Thompson Institute and Department of Chemistry and Chemical Biology, Cornell University, Ithaca, New York, United States of America; 3 Microbiology and Molecular Genetics Graduate Program, Emory University, Atlanta, Georgia, United States of America; 4 Immunology and Molecular Pathogenesis Graduate Program, Emory University, Atlanta, Georgia, United States of America; Charité-University Medicine Berlin, Germany

## Abstract

Enteropathogenic *Escherichia coli* (EPEC), enterohemorrhagic *E. coli* (EHEC) and enteroaggregative *E. coli* (EAEC) are intestinal pathogens that cause food and water-borne disease in humans. Using biochemical methods and NMR-based comparative metabolomics in conjunction with the nematode *Caenorhabditis elegans,* we developed a bioassay to identify secreted small molecules produced by these pathogens. We identified indole, indole-3-carboxaldehyde (ICA), and indole-3-acetic acid (IAA), as factors that only in combination are sufficient to kill *C. elegans*. Importantly, although lethal to *C. elegans*, these molecules downregulate several bacterial processes important for pathogenesis in mammals. These include motility, biofilm formation and production of Shiga toxins. Some pathogenic *E. coli* strains are known to contain a Locus of Enterocyte Effacement (LEE), which encodes virulence factors that cause “attaching and effacing” (A/E) lesions in mammals, including formation of actin pedestals. We found that these indole derivatives also downregulate production of LEE virulence factors and inhibit pedestal formation on mammalian cells. Finally, upon oral administration, ICA inhibited virulence and promoted survival in a lethal mouse infection model. In summary, the *C. elegans* model in conjunction with metabolomics has facilitated identification of a family of indole derivatives that broadly regulate physiology in *E. coli*, and virulence in pathogenic *strains*. These molecules may enable development of new therapeutics that interfere with bacterial small-molecule signaling.

## Introduction

Enteropathogenic *Escherichia coli* (EPEC) and enterohemorrhagic *E. coli* (EHEC) are gastrointestinal pathogens transmitted via contaminated food and water [1]. EPEC is a significant public health concern, especially in developing countries where it is a leading cause of infantile diarrhea, leading to dehydration and death [2]. EHEC is endemic to cattle in developed countries, and like EPEC, contaminates food and water. EHEC and related strains produce two Shiga toxins (STX) that cause bloody diarrhea, hemolytic uremic syndrome, kidney failure, and often death [3], [4]. There is a strong mandate to develop drugs to treat pathogenic *E. coli* infections. Antibiotics are contraindicated because they can cause lysis of the bacteria, which facilitates release of STX. Moreover, because EHEC and EAEC are endemic in cattle and fertilizers, protecting the food supply has become more difficult.

Virulence genes important for EPEC and EHEC pathogenesis are located within the 35–43 kb locus of enterocyte effacement (LEE) (Elliott et al., 1998; [5], [6]. LEE genes encode a type III secretion system and various virulence factors responsible for attaching and effacing (A/E) lesions on host intestinal epithelia (Knutton et al., 1998). A/E lesions are characterized by disruption of microvilli and formation of actin-filled protrusions or “pedestals” beneath the attached bacterium [7]. Besides the LEE, several non-LEE encoded genes also contribute to virulence, including genes encoding the two STXs of EHEC [8]. Notably, the 2011 E. coli outbreak in Germany was caused by the STX-positive enteroaggregative *E. coli* strain O104:H4 [4] [3].

Whereas considerable information is available on the regulators of the LEE and other virulence determinants, much less is known about the role of secreted small molecules in these regulatory pathways. To identify such factors, we developed a model of EPEC pathogenesis in which the bacteria kills the nematode *Caenorhabditis elegans*. *C. elegans* has been used to identify novel genes required for virulence of bacterial, fungal, and viral pathogens [9], including EPEC and EHEC [10], [11], [12], [13]. Exposure to EPEC or EHEC kills *C. elegans* within hours [10]. By contrast, brief pre-exposure to these pathogens, a process termed conditioning, induces a long-lasting protective response that allows *C. elegans* to survive a subsequent exposure to EPEC that would otherwise prove lethal [11].

To kill or condition *C. elegans*, EPEC use a secreted factor or factors, production of which requires both tryptophan in the media and the bacterial tryptophanase gene *tnaA* [10]. Tryptophanase converts tryptophan into indole, ammonia, and pyruvate, which allows EPEC, EHEC and other *E. coli* to utilize tryptophan as the sole source of carbon and nitrogen [14]. Indole is a metabolite synthesized and secreted by *E. coli* that regulates biofilm formation [15], and facilitates inter- and intracellular signaling [16] as well as adaptation to stressors [16], [17], [18].

Indole itself could be responsible for killing or conditioning *C. elegans*. However, indole is only one of many products of typtophanase and can undergo further modification in *E. coli* and related bacteria ([19], D.K., G.B., unpublished observations), raising the possibility that a variety of indole derivatives also mediate toxicity in *C. elegans.* Using biochemical methods coupled with two-dimensional NMR-based comparative metabolomics, we report here that indole and the chemically related compounds indole-3-carboxaldehyde (ICA) and indole-3-acetic acid (IAA) are produced by EPEC, EHEC, and by commensal *E. coli*. These molecules act in synergy with indole to kill and condition *C. elegans*. In addition, these molecules regulate biofilm formation, virulence, and production of Shiga toxins in pathogenic *E. coli* strains, as well as virulence in the rodent A/E pathogen *C. rodentium*, which does not contain tryptophanase nor produce indole. Finally, prior administration of ICA limits morbidity and mortality in mice infected with *C. rodentium*. Thus, the *C. elegans* bioassay has facilitated identification of secreted bacterial signaling molecules that regulate production of virulence factors and toxins important for disease in mammals. These compounds may be a means by which commensal *E. coli* suppress virulence by pathogenic strains. Moreover, these compounds may hold promise as therapeutic agents for infections caused by EPEC, EHEC, and related pathogens.

## Materials and Methods

### Bacterial Strains

The bacteria strains used in our experiments include enteropathogenic *E. coli* (EPEC) serotype O127:H6 strain E2348/69 (from B.B. Finlay; [20]), *E. coli* OP50 [21], *E. coli* P90C, and *E. coli* MG1665 (from Bernard Weiss, Emory University), and EPECΔ*tnaA* [10]. EHEC strains used include EDL933 serotype O157:H7, and 8624 serotype O157:H7, and 3023–94 serotype O104:H21. 3023–94 is derived from the outbreak of bloody diarrhea in Montana and is positive for stx2 (stx2a and stx2d variants), negative for *eae*, and positive for *ehxA* (enterohemolysin). It is also negative for *aggR* and *aatA* (pCVD432), two genes on plasmid pAA in EAEC. Enteroaggregative (EAEC) *E. coli* strains include 2011C-3493, 2009EL-2071 (O104:H4) and [3], Strains 3493 and 2071 are both positive for *stx2* (stx2a variant) and for *aggR* and *aatA*. The *C. rodentium* strain used was ATCC 51116. All bacterial strains were cultured in LB broth (Difco) to an OD_600_ of 0.8–1.0 before use.

### *C. elegans* Strains

The following *C. elegans* mutants were obtained from the *Caenorhabditis* Genetics Center: wild-type Bristol strain N2, *daf-2(e1370), sek-1(km4), spp-1(ok2703),* and *dop3(vs106)*. *daf-16(mgDf47)* was provided by S. Lee. All *C. elegans* strains were maintained on Nematode Growth Medium (NGM) under standard culturing conditions with *E. coli* OP50 as food source [22], [23].

### *C. elegans* Killing Assays

All assays were performed at 25°C essentially as described previously [10], [11]. Briefly, EPEC was cultured in Luria-Bertani (LB) broth overnight to an OD_600_ of 0.8–1.0 and 170 µL spread on 6 cm LB agar (Fisher) plates containing 2 mg/mL tryptophan (LBT plates). After incubation for 20 hours at 37°C, the LBT/EPEC plates were cooled in 25°C incubators for an hour. Young adult worms, maintained at 20°C, were collected with M9 buffer (3 g KH_2_PO_4_, 6 g Na_2_HPO_4_, 5 g NaCl, 1 mL 1 M MgSO_4_ in 1 L of water) and exposed to EPEC for 3 hours at 25°C before being transferred to OP50 on NGM plates. After 24 hours at room temperature, worms were gently prodded with a platinum wire and considered dead if they failed to respond to touch and showed no indication of pharyngeal pumping. At least 250 worms were tested for each experiment.

### Indole Killing Assays

Synthetic indole (Sigma-Aldrich) was dissolved in methanol (Sigma) to form a 1 M stock solution. Because indole readily oxidizes, solutions were made fresh and a new batch was ordered every three months. To prepare 3.5 mM indole-LB agar plates, indole was added to 100 mL of autoclaved LB agar prior to dispensing into 6 cm diameter Petri dishes (10 mL per dish). For killing assays, synchronized young adult worms maintained at 20°C were exposed to indole or methanol on the LB agar plates for 3 hours at 25°C before being transferred to OP50 on NGM plates. For experiments with indole or methanol in LB broth, worms were added to 1 ml broth in Eppendorf tubes, and gently shaken for 3 hours at 25°C, after which the worms were transferred to OP50/NGM plates. Twenty-four hours later, the percent survival was determined.

### Indole Conditioning Assays

Conditioning assays followed the general scheme described previously [11] with slight modifications. For indole pre-exposure and challenge assays**,** young adult worms were pre-exposed either to LB agar with 3.5 mM indole or in LB broth for 15 minutes (pre-exposure), washed in M9 buffer solution and transferred to OP50 on NGM plates for 3 hours (wait), and then transferred again to 3.5 mM indole on LB agar or in LB broth for 3 hours (challenge). After challenge, worms were transferred to OP50 on NGM for 24 hours (recovery) and the percent survival determined. For indole pre-exposure and EPEC challenge assays, young adult animals were pre-exposed as described above, and then challenged on LBT/EPEC plates for 3 hours before transfer to NGM/OP50. For EPEC pre-exposure and indole challenge assays, worms were pre-exposed to LBT/EPEC for 30 minutes, allowed to incubate on NGM/OP50 for 3 hours, and then challenged on 3.5 mM indole for 3 hours. For all experiments with indole, methanol controls were performed simultaneously.

### Extraction of Small Molecules from LB Agar

LBT agar plates, layered with or without 0.22 µm cellulose acetate filters, were incubated with various bacterial strains for 20 hours at 37°C. Cellulose acetate filters were used to distinguish between total amounts of molecules produced by the bacteria and molecules secreted into the agar. The filter was then removed and small molecules were extracted from the agar using a solvent mixture of dichlormethane:methanol:ethylacetate (2∶1:3) and incubation for 15 minutes in an ultrasonic water bath. Eight 10 cm plates, each with 25 mL agar, were extracted with 60 mL of solvent and reduced to dryness using a rotary evaporator. The residue was redissolved in 2 mL methanol and analyzed for the presence of indole-like structures by TLC and HPLC. The efficiency of extracting indole was determined by spiking agar plates with known indole concentrations, and extracting as above. The percent recovery based on three independent experiments was 13%.

For experiments where indole supplemented extracts, indole in LB broth was added to dried extract to a final concentration of 2 mM. The mixture was warmed for 10 minutes at 40°C, and then cooled to 25°C prior to addition of worms. After 3 hours at 25°C, worms were transferred to NGM/OP50 and the percentage survival determined 24 hours later.

### Killing and Conditioning Assays with Indole and Indole Derivatives

Stock solutions of indole (500 mM), ICA (10 mM), IAA (50 mM) and ICOOH (25 mM; all from Sigma-Aldrich) were made in methanol. To perform killing assays, young adult worms were exposed to LB broth containing 2 mM indole, 0.6 mM ICA, 0.2 mM IAA, and 0.05 mM ICOOH for 4 hours. Worms were then washed once in M9 buffer and transferred to NGM/OP50 and the percentage survival determined after 24 hours. Methanol was used as a control. For conditioning, worms were pre-exposed to the above mixtures for 30 minutes, washed once with M9 buffer and transferred to NGM/OP50 for 3 hours, and then challenged either in the mixture for 4 hours (mixture-mixture) or on LBT/EPEC plates for 3 hours (mixture-EPEC). We also pre-exposed the worms for 30 minutes to EPEC/LBT, transferred to NGM/OP50 for 3 hours, and then challenged in the mixture for four hours (EPEC-mixture).

### Thin-layer Chromatography

TLC methods optimized for indole and indole-like structures were used to analyze components of the extracts. The extract was separated on either analytical or preparative TLC plates (analytical: HPTLC plates 10×10 cm, SilicaGel 60 F_254_ (Merck); preparative: fluorescent SilicaGel G, 20×20 cm (Analtech) using toluene:acetone:chloroform (2∶1:1) as the mobile phase [24]. Plates were run in a TLC chamber for 45 min. TLC plates were coated with a fluorescent silicate, enabling detection of compounds upon excitation with UV light. A UV lamp with excitation wavelengths at 254 nm and 366 nm was used for detection. For preparative TLC plates, separated spots were tested for their ability to kill worms. Small plastic cylinders were placed on the identified spots and partially filled with LB agar. After the agar had hardened, worms were placed in the cylinders for 1 hour, after which they were moved to NGM/OP50 plates and the percentage survival determined after twenty-four hours.

### HPLC, MS and NMR Analysis

The extract was fractionated on a Shimadzu LC solution HPLC system using an Zorbax XD C18 column (Agilent) and an isocratic gradient of 50% methanol for 11 minutes at 0.5 mL/minute. For known components, standards were used to verify the identity and determine the concentration. Peak fractions were collected and analyzed by mass spectrometry (Chemical Core Facility, Emory University) and ^1^H NMR spectroscopy (Chemistry Department, Emory University) on a Varian Inova 400 (400 MHz) NMR spectrometer using CDCl_3_ as the solvent.

### Two-dimensional NMR-based Comparative Metabolomics

Extracts from EPEC or EPECΔ*tnaA* were suspended in 1 mL of 99.8% CD_3_OD. After 1 hour at 22°C, the samples were evaporated to dryness and subsequently re-suspended in 600 µL of 99.95% CD_3_OD (Cambridge Isotope Laboratories). The resulting suspensions were centrifuged at 4,400 rpm for 2 minutes, and the supernatants were transferred to 5 mm NMR tubes. ^1^H and high-resolution dqfCOSY NMR spectra (acquisition time 0.6 second, sweep width 10 ppm, number of increments 600) were recorded on a Varian INOVA 600 NMR (600 MHz for ^1^H, 151 MHz for ^13^C) with a 5 mm inverse-detection HCN probe. dqfCOSY spectra were processed using Topspin (Bruker) and zero filled 8 k and 4 k points in the direct and indirect dimensions, respectively. Differential analysis of dqf-COSY spectra was adapted from previous work [25], [26]. In short, dqfCOSY spectra were overlaid using Topspin and differentially present peaks were further characterized.

HPLC-MS analysis was conducted using an HPLC system with a diode array detector operating at wavelengths of 210, 230, and 260 nm and connected to a Quattro II spectrometer (Micromass/Waters) operated in positive-ion (ESI+) or negative-ion electrospray ionization (ESI-) mode. Data acquisition and processing for the HPLC-MS was controlled by Waters MassLynx software. Reverse phase chromatography using an eclipse XDB-C18 column (Agilent) was performed using a 95% to 0% H_2_O (0.1% acetic acid)/acetonitrile gradient. Indole, ICA, ICOOH, IAA standards (Sigma Aldrich, St. Louis MO) were used for structural confirmation and to determine the concentration of these compounds in the bacterial extracts based on UV absorbance.

### Western Analysis of Tir/LEE Expression

Ler, Tir, and EspA were analyzed by Western blotting as described [27]. Proteins were electroblotted onto a PVDF membrane and probed with either mouse DnaK mAb (1∶10,000; Enzo), Ler pAb (1∶2,500; provided by I. Rosenshine), EspA pAb (1∶50,000; provided by J. Kaper), or Tir pAb (1∶50,000; provided by J. Kaper). Experiments were repeated at least three times.

### Pedestal Assays, Cell Culture and Immunofluorescence Microscopy

3T3 cells were maintained and passaged under standard culture conditions in DMEM supplemented with 10% fetal bovine serum (cDMEM). Pedestal formation by EPEC and *C. rodentium* on 3T3 cells was performed as described previously [28] with some modifications. Briefly, 3T3 cells were grown to approximately 70% confluence on glass coverslips in 24-well microtiter plates in cDMEM. Prior to infection the cDMEM was aspirated and replaced with serum free DMEM. Bacterial cultures were grown overnight without shaking at 37°C to an OD_600_ of ∼1.0 in LB. Cells were infected with equal number of bacteria as determined by OD_600_. Infections were allowed to proceed for 5 hours after which the cells were washed with PBS and fixed, stained, and imaged as described previously [28]. Pedestal assays were repeated at least three times from separate experimental samples with each sample being assayed in duplicate. The results presented are representative of three independent experiments.

### Shiga Toxin Cytotoxicity Assay

STX assays were performed essentially as described previously [29]. Briefly, Vero cells were plated at 10^4^ cells/well in a 96 well dish. Lysate from bacteria grown to an OD_600_ of 0.2 in the presence of indole derivatives was added to the wells in serial 10 fold dilutions. After 48 hours, the supernatant was removed and the cells were fixed in 2% formalin, and stained with 0.13% w/v crystal violet solution in 5% ethanol and 2% formalin. After drying, crystal violet was dissolved in 200 µl 50% ethanol and the OD_595_ was plotted as a function of the dilution.

### Biofilm and Motility Assays

Stationary phase EHEC or EAEC cultures were diluted 1∶100 with DMEM +0.2% glucose and various indole derivatives added. Bacterial solutions were added to tissue culture coated 96 well plates (Corning) and incubated at 37°C for 48 hours. Biofilm formation was visualized by crystal violet staining as described above, and formation or loss of biofilm was measured at OD_595_. Motility in soft agar was measured as described previously [27].

### *In vivo* Infections and Analysis

MyD88^−/−^ mice on a C57BL/6 background were the generous gift of David Underhill, and were originally generated in the lab of Shizuo Akira (see also [30]). Animal care was provided in accordance with protocols approved by the Institutional Animal Care and Use Committee of Emory University. *C. rodentium* were prepared by overnight culturing (12 to 16 hours) at 37°C in LB without shaking. Cultures were harvested by centrifugation and resuspended in a 20% sucrose solution. For infections, drinking water was replaced with *C. rodentium* suspension overnight. The volume of suspension was measured before and after administration, and the number of bacteria in the inoculum was calculated following retrospective plating. The average dosage was 2×10^8^ CFU/mouse. Survival of infected mice and changes in body weight were monitored daily. Mice losing at least 15% of their original weight were euthanized. For histological studies, colons, livers, and spleens were removed from infected mice, fixed in 10% formalin, and embedded in paraffin. Sections (5 µm) were cut and stained with H&E by the Translational Research Lab at Emory University. To measure the CFU of *C. rodentium,* tissue samples of colon, liver and spleen weighing ∼0.1 to 0.3 g were homogenized at low speed with a Tissuemizer (Fisher Scientific) in 1 mL of PBS. The lysate was plated on MacConkey agar plates at various dilutions, and *C. rodentium* colonies were recognized as pink with a white rim as previously described [30], [31]. Pink colonies were counted after 20 hours of incubation at 37°C to determine the CFU per gram of tissue. A total of 45 mice were used to conduct the experiments; all mice were age-matched.

### Statistical Analysis

Methods have been described in detail elsewhere [11]. Briefly, for *C. elegans* survival experiments, we used an ANOVA and a test for linear trend in survival over time. The comparisons were highly significant. Mean survival +/− SEM is shown for the indicated time point. For conditioning experiments, we used ANCOVA to remove the effects of pre-existing mutant differences, and ensure that mutants are starting out approximately equal, on average, with respect to all factors that might be pertinent to how well they are likely to respond to the conditioning paradigms. Such a correction is useful because individual differences in conditioning displayed by a particular mutant could potentially be correlated with survival rate. For these experiments, 95% confidence intervals are shown. Thus, significance at the 0.05 level is achieved between conditions or mutants when confidence intervals do not overlap. For mortality curves with mice, a Kaplan Meyer test determined statistical significance. For other experiments in Figures 5–7, a Student’s *t* test assessed statistical significance. Results were considered significant if the *p* value was less than 0.01. Statisitcal significance relative to the control is indicated by an *.

## Results

### Identification of Indole as a *C. elegans* Killing Factor

**Figure 1:**
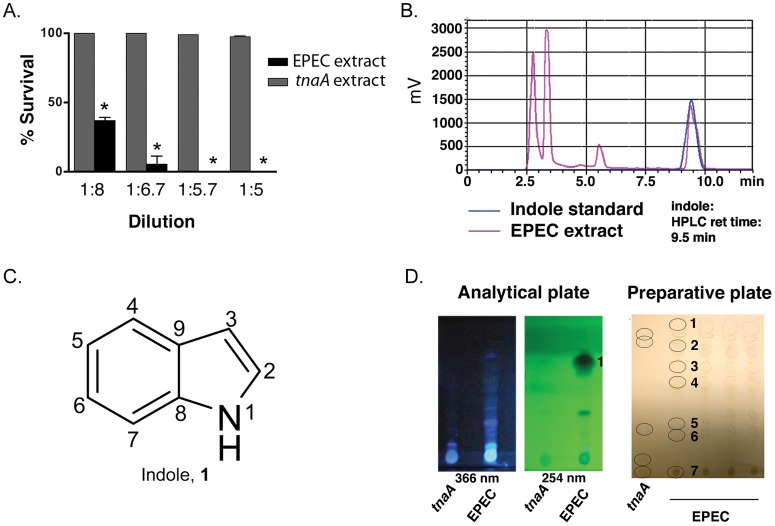
Identification of extract components that kill *C. elegans*. (**a**) Effect on *C. elegans* survival of dilutions of extracts derived from agar plates on which EPEC or EPECΔ*tnaA* were grown. Extracts comprise only secreted bacterial factors because bacteria were grown on 0.2 µm nitrocellulose filters and then discarded prior to extraction of small molecules from the agar. EPEC, but not EPECΔ*tnaA,* extracts kill *C. elegans*. Mean +/− SEM are shown; * corresponds to p<0.001 with respect to control at each dilution. (**b**) HPLC profile of EPEC extracts or a synthetic indole standard. Note that the extract peak on the right overlaps with one seen with synthetic indole. The second peak from the left corresponds to tryptophan, and the third to ICA (see Fig. S3a). (**c**) Structure of indole. (**d**) Images of analytical and preparative TLC plates used for separation of EPEC and EPECΔ*tnaA* extracts. The preparative plate was imaged under visible light, and numbers and circles indicate spots, evident under UV illumination, onto which agar filled cylinders were placed and to which *C. elegans* were added. Only spot #1 killed *C. elegans*.

To identify bacterial factors responsible for killing *C. elegans*, EPEC was cultured overnight on nitrocellulose filters atop LB agar plates with added tryptophan (LBT), and then discarded with the filters. After extraction of the agar with organic solvents, a crude extract was generated. As shown in Fig. 1a, the extract at dilutions up to 1∶6 killed all animals within 2 hours. By contrast, an extract from cultured EPECΔ*tnaA* was without effect even at dilutions as high as 1∶2. HPLC analysis of the extract yielded several peaks (pink line, Fig. 1b), one of which had the same retention time as commercially available indole (blue line, Fig. 1b). This peak was confirmed as indole (Fig. 1c; Fig. S1 **,** compound **1**) by HPLC-MS (not shown), with the most abundant peak at 118 Da. Because of the requirement for tryptophanase activity in killing by EPEC, the extract was fractionated using a TLC separation method optimized for indole-like compounds. Spots containing indole-like compounds were recognized by their fluorescence upon illumination with UV_366_ or UV_254_ (Fig. 1d). To assess killing activity of the indole-like molecules, conical cylinders were placed over each spot and partially filled with LB agar. After the agar had solidified, *C. elegans* were added to the agar. Cessation of movement was evident within minutes for the spot with the highest Rf (Spot #1, Fig. 1d), and removal of animals one hour later to NGM/OP50 for twenty-four hours confirmed that all animals had died. No other spots proved toxic. Notably, extracts from EPECΔ*tnaA* did not show a spot with the Rf value of spot #1. Moreover, HPLC analysis indicated that Spot #1 eluted with the same retention time as commercially available indole (not shown). These data suggest that indole in the extract is toxic to *C. elegans*.

**Figure 2:**
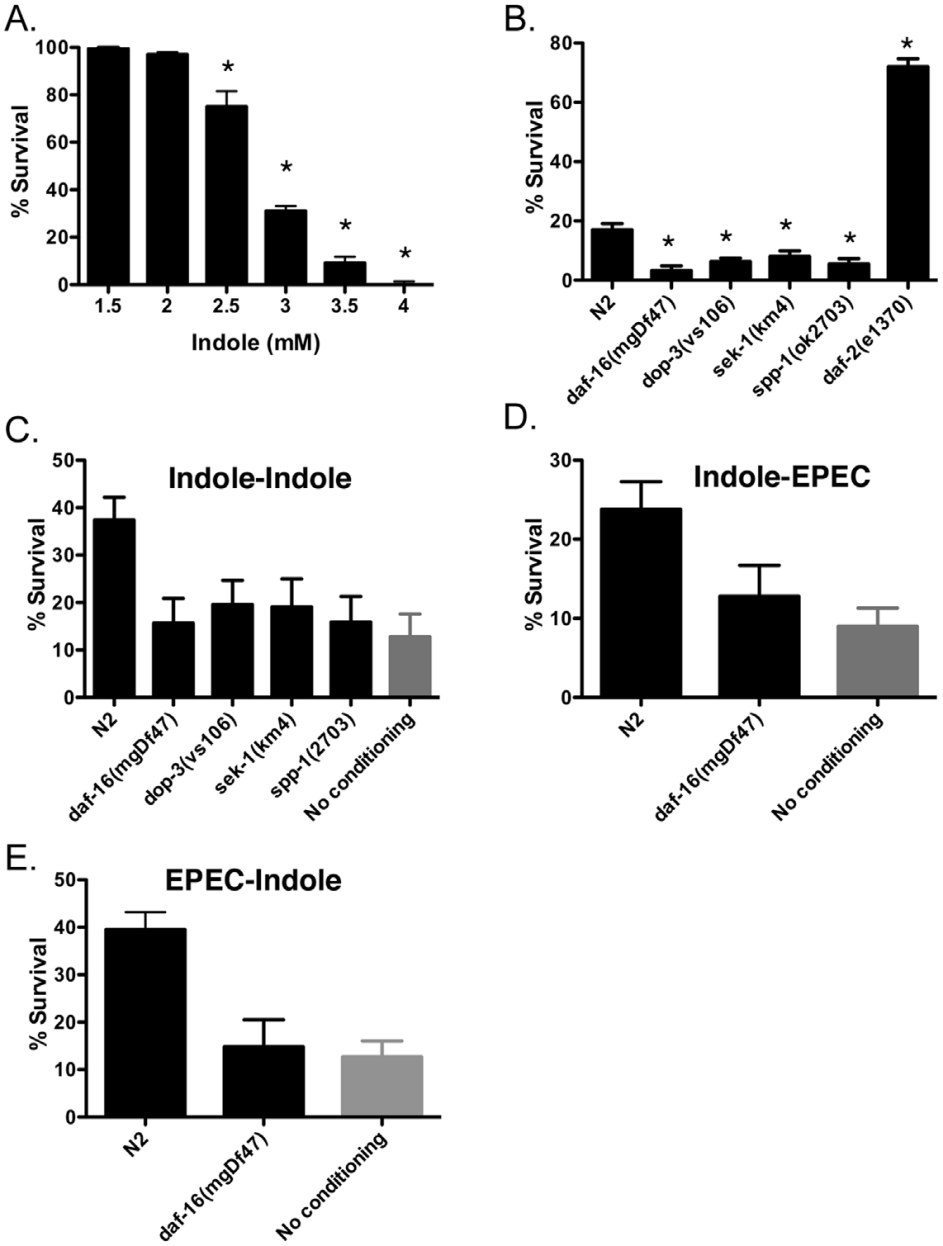
Effects of synthetic indole on *C. elegans*. (**a**) Effects on survival of *C. elegans* N2 upon exposure to various concentrations of synthetic indole in LB broth for 3 hours. (**b**) Effects on survival of *C. elegans* N2 and various mutants upon exposure to 3.5 mM synthetic indole in LB broth for 3 hours. In a-b, mean +/− SEM are shown; * corresponds to p<0.01 with respect to controls (1.5 mM in A; N2 in b). (**c**) Effects of pre-exposure of N2 or various mutants to 3.5 mM indole for 15 minutes followed by a 3 hour waiting period on OP50, and then a 3 hour challenge with 3.5 mM indole. The grey bar indicates the percent survival without conditioning. (**d,e**) Effects of pre-exposure of N2 or *daf-16(mgDF47)* to indole (15 minutes) or to EPEC (30 minutes) followed by a 3 hour waiting period on OP50, and then a 3 hour challenge with either EPEC/LBT (**d**) or 3.5 mM indole (**e**). In both cases, only N2 was conditionable. In c-e, mean +/−95% confidence intervals are shown. Lack of overlapping error bars indicates significance at the 5% level.

To confirm this possibility, we assessed whether synthetic indole affected survival of *C. elegans* by exposing N2 worms to LB broth containing indole at concentrations ranging from 1.5 mM to 4 mM for 3 hours. More than 95% of the animals survived exposure to indole concentrations 2 mM or lower, whereas less than 10% survived exposure to concentrations greater than 3.5 mM (Fig. 2a). Methanol, the solvent used to dissolve indole, was without effect (not shown; see also Fig. S3b). This effect was also evident in solid media because 3.5 mM indole on agar plates likewise killed N2 worms in 3 hours (Fig. S2a). Collectively, these data confirm that indole is sufficient to kill *C. elegans*, in accordance with the requirement for tryptophanase activity for EPEC pathogenicity [10].

The identification of indole as a toxin raised the possibility that other *E. coli* strains might also kill *C. elegans*. Whereas EHEC and certain strains of commensal *E. coli* (e.g. MG1655) killed *C. elegans* similarly to EPEC, other K12 strains were without effect (e.g. OP50, P90C; Fig. S2b). Although all these strains had similar levels of tryptophanase activity ([10] and not shown), they appeared to secrete different levels of indole (Fig. S2c). Some differences were evident when bacteria were grown on different media (e.g. ECD vs. LBT agar; data not shown; see also [10]). Nevertheless, the amount of secreted indole was highly correlated with the capacity to kill *C. elegans*, in accordance with the concentration-dependent toxicity of synthetic indole (Fig. 2a).

### Indole-dependent Killing Depends Upon Genes Controlling Dopamine Signaling, Innate Immunity, and Longevity

We reported previously that *C. elegans* genes controlling dopaminergic signaling, innate immunity and lifespan mediate protection from EPEC [10], [11]. To determine whether these genes were similarly required for protection against indole, we assessed the effects of this compound on *C. elegans* strains containing mutations in the dopaminergic receptor *dop-3* (*dop-3(vs106*)), the insulin receptor *daf-2* (*daf-2(e1370*)), which mediates longevity [32], the *daf-2* effectors *daf-16* (*daf-16(mgDf47*)) and spp-1 (*spp-1(ok2703))*, which abrogate the effects of *daf-2, and sek-1* (*sek(km4)),* a MAPKK that regulates innate immunity [33]. As with EPEC, *daf-16(mgDF47)*, *sek-1*(km4), *dop-3(vs106)* and *spp-1(ok2703)* appeared more sensitive to indole than N2, indicating that these pathways protect worms from the effects of indole, whereas *daf-2(e1370)* was resistant (Fig. 2b, broth; Fig. S2a, agar), a pattern identical to that seen with EPEC [11].

### Indole Conditions *C. elegans*

Brief pre-exposure of *C. elegans* to EPEC conditions animals and permits survival from a subsequent exposure that would otherwise prove lethal [11]. The effect is induced by toxic or nontoxic factors within EPEC, and is mediated by dopaminergic neurons as well as innate immune and longevity genes [11]. Analogous to EPEC, pre-exposure of wild type *C. elegans* (N2) for 15 minutes to indole in broth increased survival by 3 fold upon subsequent exposure to 3.5 mM indole in broth for 3 hours (indole-indole; Fig. 2c). Likewise, pre-exposure of N2 to indole in LB agar provided protection against a subsequent challenge with indole in LB agar (Fig. S2d).

We next assessed whether the same pathways were used for conditioning with indole and EPEC [11]. In accordance with previous results [11], *dop-3(vs106)*, *sek-1(km4)*, *daf-16(mgDf47)* and *spp-1(ok2703)* animals were not conditionable with indole against subsequent challenge with indole (Fig. 2c; Fig. S2e). Moreover, pre-exposure to indole conditioned wild type but not *daf-16(mgDf47)* animals to survive a subsequent EPEC exposure (Fig. 2d). Likewise, pre-exposure to EPEC conditioned N2 but not *daf-16(mgDf47)* animals to survive a subsequent exposure to indole (Fig. 2e). Thus, the same gene products appear to mediate conditioning by indole and EPEC.

### Additional Factor(s) in the Extract Facilitate Killing by Indole

**Figure 3:**
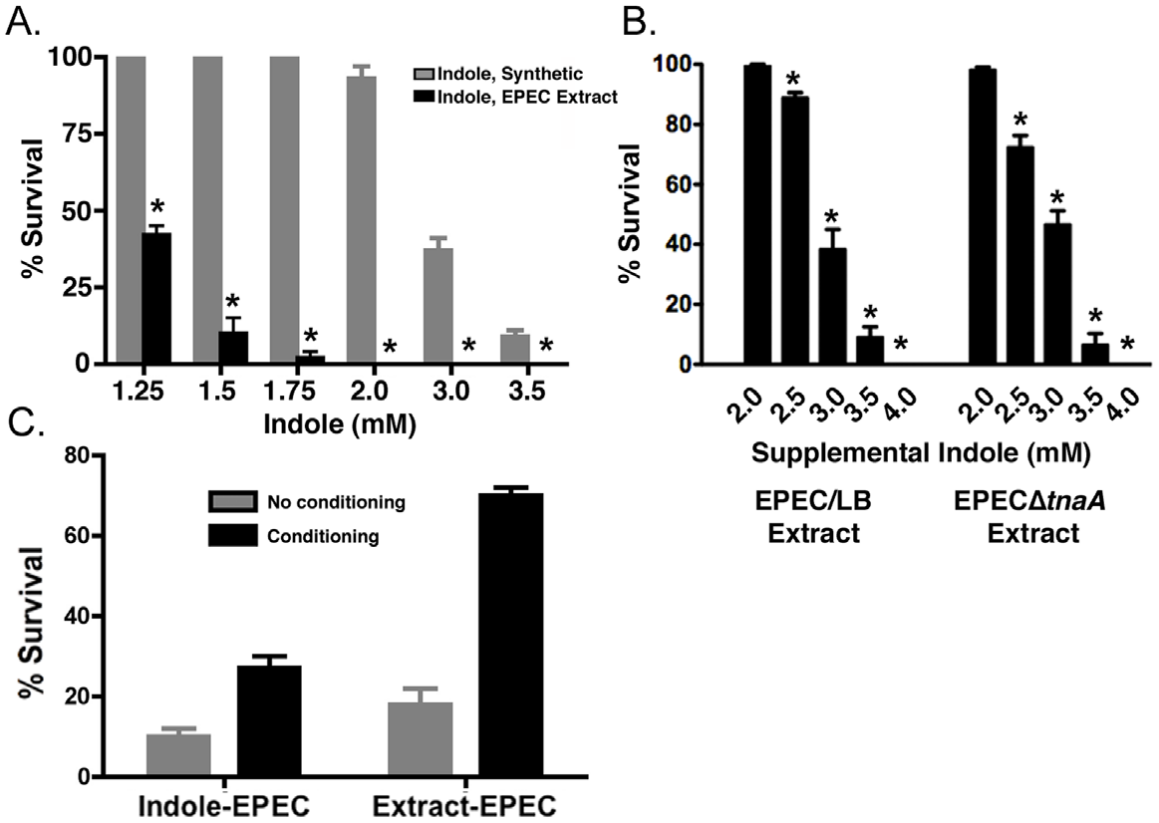
Effects of synthetic indole or extract on killing and conditioning of *C. elegans*. (**a**) Comparison of the effect of synthetic indole or of EPEC agar extracts in which the indole concentration has been estimated based on HPLC measurements of the indole concentration in identically prepared samples. Note that the LD_90_ for synthetic indole is 3.5 mM, but that the extract kills even at indole concentrations of 1.5 mM, suggesting that additional factors in the extract contribute to killing. Mean +/− SEM are shown; * corresponds to p<0.001 with respect to control at each dilution. (**b**) Tryptophan and tryptophanase are required for production of additional killing factors. N2 worms were exposed either to EPEC extracts from LB plates lacking tryptophan or to EPECΔ*tnaA* extracts. Significant killing was evident in either condition only when synthetic indole was added at concentrations greater than 3 mM. Mean +/− SEM are shown; * corresponds to p<0.01 with respect to control at each dilution. (**c**) Comparison of the effects of conditioning *C. elegans* with either 3.5 mM indole or with extract derived from EPEC/LBT plates, and challenging with EPEC. Note that extract conditions 2-fold better than indole. Mean +/−95% confidence intervals are shown. Lack of overlapping error bars indicates significance at the 5% level.

We next determined whether the extract from EPEC plates contained indole concentrations that corresponded to those required to kill or condition *C. elegans*. We first measured the yield of indole in the extraction protocol by using EPECΔ*tnaA* plates spiked with known amounts of indole. On average, the protocol yielded a 13% recovery (n = 4). We next calculated the concentration of indole in the extract dilutions. As shown in Fig. 3a, the extract killed *C. elegans* at lower apparent concentrations of indole than were achieved with synthetic indole. Comparison of the LD_90_ for synthetic indole (∼3.50 mM; Fig. 2a) with the LD_90_ for the indole in the extract (∼1.5 mM) revealed a 2.3-fold difference (Fig. 3a). Thus, the apparent concentration of indole in the extract alone did not appear sufficient to kill *C. elegans*, raising the possibility that additional factors in the extract might have killing activity or, alternatively, potentiate the effects of indole.

The additional factor(s) was not capable of killing on its own because no killing was evident with EPEC extracts in which the indole was removed upon fractionation by HPLC (data not shown). Thus, the additional factors appeared to enhance the killing capacity of indole, thereby lowering its critical killing concentration when administered together with lower concentrations of indole.

We next assessed whether tryptophan or tryptophanase were required for production of the additional factor(s). To do this, EPEC was grown on LB agar plates without tryptophan, and an extract lacking indole was prepared. Synthetic indole was then added to the extract at different concentrations. As shown in Fig. 3b, the supplemented extract was only capable of killing *C. elegans* when the concentration of supplemental indole exceeded 3.0 mM, indicating that production of the additional factor likely depended upon tryptophan. Moreover, extracts from EPECΔ*tnaA* mutant only killed when supplemented with indole at concentrations greater than 3.0 mM, suggesting that production of sufficient levels of the additional factor also depended upon tryptophanase activity (Fig. 3b). In accordance with these data, the EPEC extract conditioned better than indole alone (Fig. 3c). These data suggest that tryptophan and tryptophanase are required for production of additional tryptophan-derived small molecule(s) that contribute(s) to EPEC pathogenicity.

### Identification of Additional Factors in the EPEC Extract

**Figure 4:**
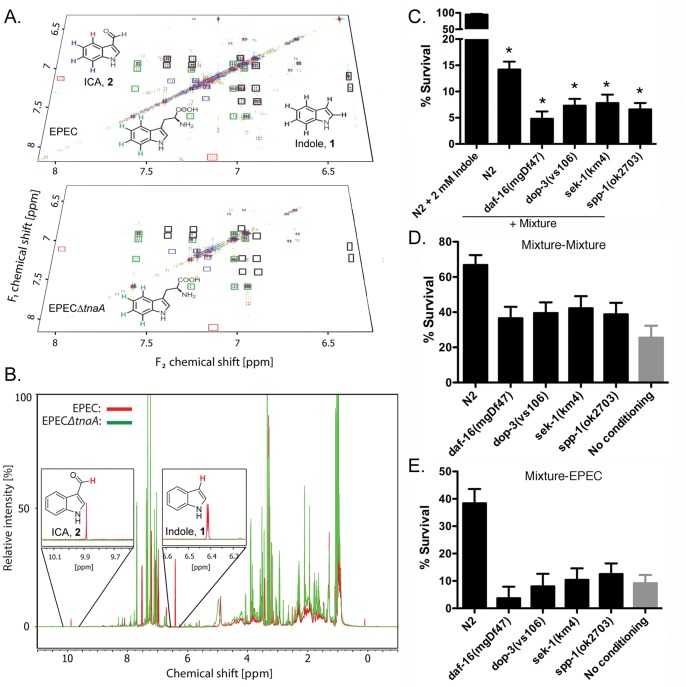
Identification of indole derivatives by two-dimensional NMR-based comparative metabolomics. (**a**) DANS overlay of the aromatic region of a dqfCOSY spectra obtained for the EPEC and EPECΔ*tnaA* extracts highlights differentially present compounds in the EPEC extract. Crosspeaks representing colored protons in the shown indole structures are highlighted by correspondingly colored boxes. For example, the crosspeaks marked with a red box corresponds to a de-shielded protons of ICA, which is present in the EPEC spectrum but absent in the EPECΔ*tnaA* spectrum. (**b**) Overlay of ^1^H-NMR spectra from the EPEC extract (red) and the EPECΔ*tnaA* (green). Signals representing indole (zoom of 6.4 ppm) and the aldehyde proton in ICA (at 9.9 ppm) are present in the EPEC spectrum but absent in the EPECΔ*tnaA* spectrum. (**c**) Reconstitution of killing with 2 mM indole in combination with 0.6 mM ICA and 0.2 mM IAA. Mean +/− SEM are shown; * corresponds to p<0.01 with respect to N2. (**d,e**) Pre-exposure of N2 or various mutants to the indole/ICA/IAA mixture for 30 minutes followed by a 3 hour waiting period on OP50, and then a 3 hour challenge with either the mixture (**d**) or EPEC (**e**). Note that killing and conditioning with the mixture closely resembles that seen with EPEC, and utilizes the same gene products in *C. elegans*. In d-e, mean +/−95% confidence intervals are shown. Lack of overlapping error bars indicates significance at the 5% level.

To identify the additional factor(s), we compared extracts from EPEC or EPEC*ΔtnaA* via differential analysis by 2D NMR spectroscopy (DANS) and HPLC-mass spectroscopy-based comparative metabolomics (Fig. 4a; [25], [26]). EPEC and EPEC*ΔtnaA* extracts were used to acquire double quantum filtered correlation spectroscopy (dqf-COSY) spectra. These spectra were analyzed using the DANS method, which highlights cross peaks stemming from metabolites differentially present in one sample and absent in another (Fig. 4a). DANS revealed a series of strong NMR signals representing indole in the EPEC spectra (Fig. 4b), and as expected indole was absent in the EPEC*ΔtnaA* spectrum. In addition, cross peaks representing tryptophan were more prominent in the EPEC*ΔtnaA* sample. In addition we detected another strong set of indole-like signals in the EPEC spectrum that was absent from EPEC*ΔtnaA* spectrum, and therefore represented an additional tryptophanase-dependent indole derivative. Detailed spectral analysis indicated that this metabolite was either indole-3-carboxaldehyde (ICA; **2**, Fig. S1) or indole-3-carboxylic acid (ICOOH, **4**), based on the characteristically high chemical shift of a crosspeak representing the proton in position 4 of the indole ring system (Fig. 4a). Additional analysis of the ^1^H-NMR spectra of EPEC and EPEC*ΔtnaA* extracts revealed an aldehyde proton in the EPEC spectrum that was absent from the EPEC*ΔtnaA* spectrum (Fig. 4b), providing further evidence that extracts from EPEC, but not EPEC*ΔtnaA*, contain ICA. Considering the possibility that ICOOH crosspeaks could have been obscured by the more abundant ICA, we further compared the EPEC and EPEC*ΔtnaA* extracts via HPLC-MS, which confirmed the presence of ICA in addition to smaller amounts of ICOOH and indole-3-acetic acid (IAA, **3**) in EPEC extracts. Indole, ICA and ICOOH were absent in EPEC*ΔtnaA* extracts, whereas IAA was also present in EPEC*ΔtnaA* extracts, although in smaller amounts than in EPEC. Using synthetic standards of known concentrations, UV absorbance at 260 nM was used to calculate the relative concentration of indole:ICA:IAA:ICOOH: in the EPEC extracts as 7∶1:0.3∶0.1 (Fig. S3a).

### Indole Derivatives Facilitate Killing and Conditioning of *C. elegans*

We next assessed the effects of the identified indole derivatives on killing and conditioning. Like indole at 2 mM, neither ICA, nor IAA, nor ICOOH killed *C. elegans*, even when administered at 3.5 mM, the highest concentration tested (Fig. S3b). However, killing was evident with 2 mM indole in combination with ICA and IAA, the latter at concentrations similar to those found in the extract (0.6 mM and 0.2 mM, respectively; Fig. 4c), though over a slightly longer time course (4 hours) than with EPEC or extract. Other ratios proved less optimal, and addition of ICOOH to the mixture was without effect (data not shown). Likewise, the mixture without indole was also without effect (Fig. S3b). Exposure of N2 animals to the indole carrier, methanol, was also without effect (Fig. S3b). Mutants in the dopamine, innate immunity and longevity pathways were more sensitive to the mixture compared to the wild type (Fig. 4c).

In conditioning experiments, pre-exposure to the mixture increased survival of N2 worms upon challenge with either the mixture (Fig. 4d) or EPEC (Fig. 4e). Individual components of the mixture induced only marginal conditioning upon challenge with EPEC. Thus, pre-exposure to ICA or indole increased survival upon subsequent exposure to EPEC by less than 2-fold, whereas IAA was less effective (Fig. S3c). Likewise, pre-exposure to EPEC induced conditioned survival upon exposure to the mixture (not shown). As with EPEC, mutants in the dopamine, innate immunity or longevity pathways were not conditionable with the mixture (Figs. 4d,e). Together, these data suggest that the mixture of indole and its derivatives generally recapitulates the effects of EPEC on both killing and conditioning.

Our identification of indole derivatives produced by commensal and pathogenic E. coli strains was based on their toxic effects in a *C. elegans* bioassay. In an effort to assess the physiological relevance of these molecules, we next explored the effects of these molecules on the bacteria themselves, and in a mammalian infection model.

### Indole Derivatives Regulate Virulence in EPEC

We showed previously that 50 µM indole activated virulence gene expression in EPEC [10]. Thus, we next assessed whether indole derivatives might likewise regulate virulence of EPEC, EHEC and *C. rodentium*, an A/E pathogen that neither contains *tnaA* nor produces indole nor ICA. Virulence was assessed by expression of the LEE-transcriptional regulator Ler, by expression of the LEE encoded virulence factor Tir, or by the extension of actin-filled membraneous protrusions beneath bacteria adhering to cultured fibroblasts, called pedestals [28]. Formation of pedestals requires Ler, a functional Type III Secretion System, and various other LEE-encoded effectors and virulence factors including Tir [34].

**Figure 5:**
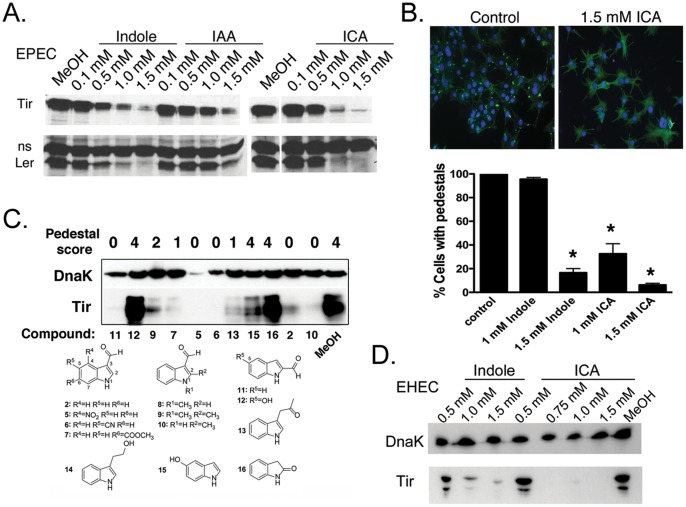
Effects of indole and indole derivatives on LEE gene expression and pedestals. (**a**) Western analysis of Tir and Ler expression in EPEC treated with various concentrations of indole, ICA or IAA. The non-specific band recognized by the Ler pAb served as a loading control. In some experiments, DnaK pAb was used as a loading control and gave identical results (see Fig. S4a). (**b**) **Above**. Images of 3T3 cells infected for 5 hours with EPEC and treated with 1.5 mM ICA or left untreated. Cells were stained with FITC Phalloidin to recognize actin and DAPI to recognize DNA. At this magnification, pedestals are seen as bright green “dots.” Magnification 200×. **Below**. Quantitation of pedestal formation in 3T3 cells infected with EPEC in the presence of indole or ICA at various concentrations. Mean +/− SEM are shown; * corresponds to p<0.01 with respect to untreated control. (**c**) Western analysis of Tir and DnaK expression in EPEC treated with various synthetic indole derivatives each at 1.5 mM. High Tir levels correlated with pedestal formation, and low DnaK levels correlated with slower growth. Pedestal scoring scale ranged from 0 to 4 (0 corresponds to no pedestals and 4 to pedestal formation as in control). Structures and growth curves are presented in Figs. S1 and S4b, respectively. (**d**) Western analysis of Tir expression in EHEC O157:H7, strain EDL933 treated with various concentrations of indole or ICA.

The effects of indole and its derivatives on Ler and Tir of EPEC appeared to be concentration dependent. At low concentrations (50 µM), indole (not shown) and ICA (Fig. S4a) induced expression of Tir from EPEC, in agreement with previous studies [10], [35]. However, at higher concentrations (>1 mM), indole, ICA, and IAA inhibited expression of LEE genes (Fig. 5a), with the effects of ICA more potent than those of indole or IAA. Maximal effects of indole and ICA were evident after 5 hours (Fig. 5a). Neither indole nor ICA affected growth of the bacteria (Fig. S4b), even when delivered in combination (data not shown). Subsequent experiments focused on ICA because it appeared most potent amongst the derivatives.

In accordance with these results, ICA at 1.5 mM reduced pedestals formed by EPEC on 3T3 cells by 7 fold at 5 hours p.i. (Fig. 5b). Indole at 1 mM was without effect, though pedestals were inhibited at higher concentrations (1.5 mM; Fig. 5b). Together, these data suggest that indole and its natural derivatives regulate LEE expression and pedestal formation in EPEC.

The apparent differences between the sensitivity of LEE expression to indole, ICA and IAA lead us to test a set of commercially available synthetic derivatives with substitutions or additions at various positions on the benzene (**5**, **6**, **7**, **12**, **15;** Fig. S1) or pyrrole (**8**, **9**, **10**, **11**, **12**, **13**, **14**, **16**) rings, so as to determine a structure-activity profile (Fig. S1). The presence of a carboxaldehyde group at position 2 (**11**, **12**) or 3 (**2**, **5**, **6**, **7**, **8**, **9**, **10**) appeared essential for inhibiting Tir expression and pedestal formation (Fig. 5C; Fig. S1). Carbonitrile groups (**6**) or carboxylic acid methyl ester at position 5 (not shown) and 6 (**7**), respectively, potentiated the inhibitory effects of ICA, whereas a hydroxyl group at position 5 in addition to the carboxaldehyde group at position 2 (**12**) abrogated this effect. Finally, a nitro group at position 4 (**5**) resulted in antibacterial activity, as indicated by reduced DnaK levels and reduced growth rates in LB broth (Fig. 5c; Fig. S4b). Compared to unsubstituted ICA, several other substitutions or additions, particularly on the benzene ring, were without additional effect, whereas others were more potent compared to unsubstituted ICA (**6**). Together, these data suggest that a carboxaldehyde group at the position 2 or 3 is required for effects of ICA on virulence, and that additional functional groups can potentiate or abrogate the effects of ICA, or even cause suppression of growth.

### Indole Derivatives Regulate Virulence in EHEC

We next assessed effects of indole derivatives on various other pathogenic *E. coli* strains. While all these strains expressed Shiga toxins (STXs), some contained the LEE (EDL933, 8624), and are classified as EHEC strains, whereas others did not (2071 and 3493), or lacked key genes required for pedestal formation (3023). 2071 and 3493 are classified as enteroaggregative *E. coli* (EAEC) strains because they contain *aggR* and *aatA*, and are human isolates from recent outbreaks in the Republic of Georgia, and Germany [4] [3].

As with EPEC, indole and ICA suppressed expression of Tir in EHEC (Fig. 5d) and secretion of another LEE effector EspA (Fig. S4c), and reduced pedestal formation (not shown). The effect of indole or ICA on Ler could not be assessed because Ler was below detectable levels in this strain, as reported previously [35]. None of these molecules affected the growth rate of the bacteria (Fig. S4b). Thus, EPEC and EHEC appear to share LEE regulatory pathways that are sensitive to indole and its derivatives.

### Indole Derivatives Regulate Shiga Toxin Production in EHEC and EAEC

**Figure 6:**
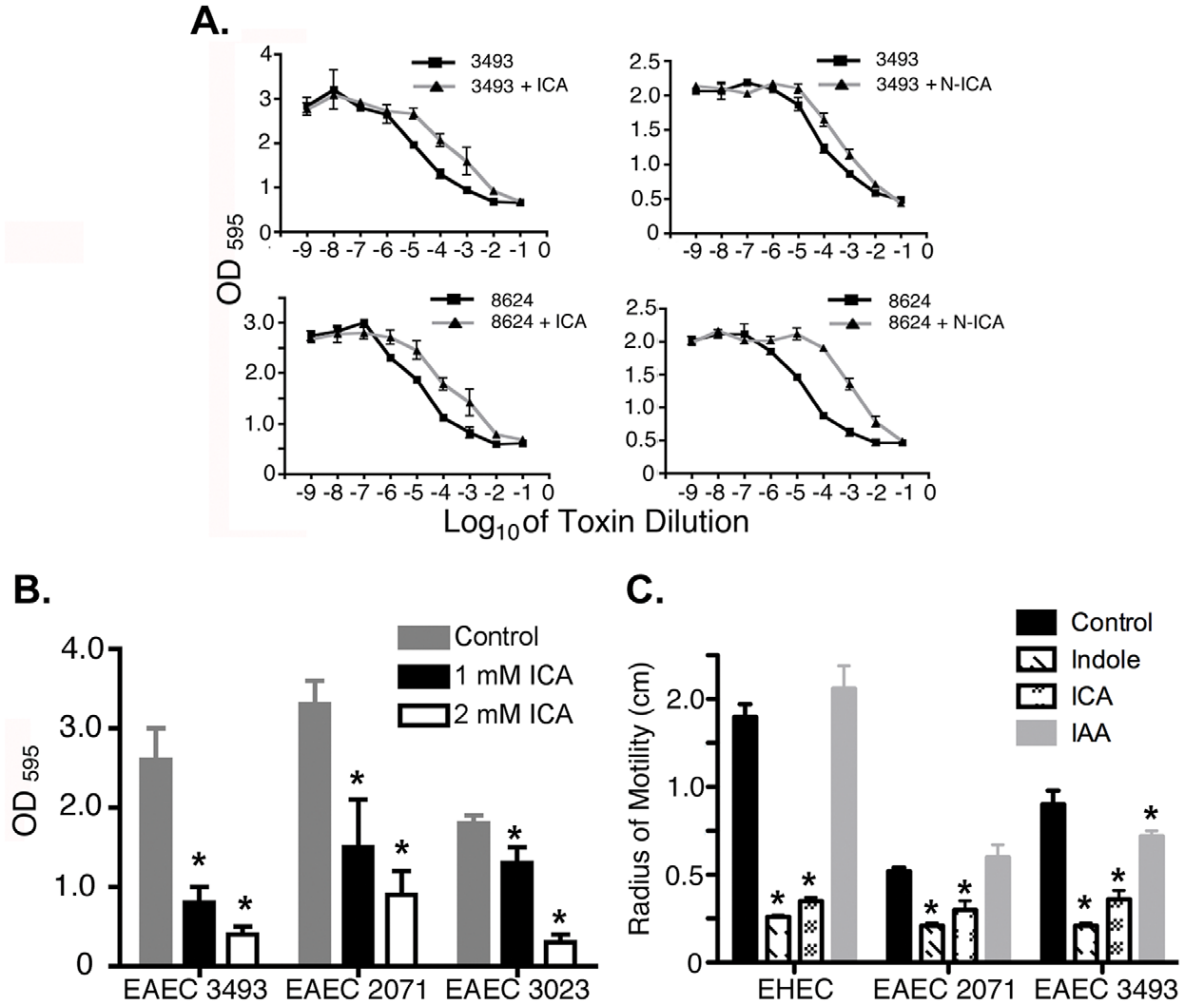
Indole derivatives inhibit Shiga toxin production, biofilm and motility in EHEC and EAEC. (**a**) Effects of ICA or the synthetic analog 4-nitroindole-3-carboxaldehyde (N-ICA) on Shiga toxin production by EHEC 8624 or EAEC 3493. Cytotoxic effect on vero cells was used as a measure of Shiga toxin production. (**b**) Effects of ICA on biofilm in EAEC 3493, EAEC 2071, and EHEC 3023. (**c**) Effects of Indole, ICA, and IAA on motility in soft agar of EAEC 3493, EAEC 2071, and EHEC O157:H7 EDL933. For b-c, mean +/− SEM are shown; * corresponds to p<0.01 with respect to untreated control.

Release of STX1 and STX2 by EHEC and EAEC strains causes hemorrhagic colitis and hemolytic uremic syndrome, and is highly correlated with mortality [4]. We next assessed the effects of indole derivatives on STX production in EHEC 8624 and EAEC 3493, which produce STX2. ICA reduced by 10 fold the IC_50_ for cytotoxic effects on mammalian cells of STX produced by both EHEC and EAEC (Fig. 6a). Notably, other indole derivatives, such as 4-nitroindole-3-carboxaldehyde (N-ICA; **5**), caused 100-fold reductions in IC_50_ for cytotoxic effects of STX in EHEC O157:H7 8624 (Fig. 6a). Although **5** slowed bacterial growth (Fig. S4b), this property appeared to be independent of its effect on toxin production. Thus, a 10–100 fold reduction in cytotoxicity was evident when the bacteria were grown with or without **5** for the same amount of time and the amount of lysate added in the cytotoxicity assay normalized based on OD_600_. A similar fold reduction was evident when cultures with or without **5** were grown to the same OD_600_, or when **5** was added after mid-log phase and the bacteria allowed to grow until they reached stationary phase albeit at a slower rate (data not shown). Collectively, these data indicate that indole, ICA or its derivatives reduce virulence and production of STXs *in vitro* in various EPEC, EHEC, and EAEC strains, including human isolates from recent outbreaks.

### Indole Derivatives Reduce Biofilm Formation and Motility in EHEC and EAEC

For EHEC 3023, and EAEC 2071 and 3493, we also assessed the effects of ICA on the capacity to form a biofilm, a property associated with virulence in some bacterial pathogens [36]. As shown in Fig. 6B, ICA suppressed formation of biofilm, with the strongest effect evident in EAEC 3493. In addition to biofilm, indole and ICA also reduced motility in soft agar in all EHEC and EAEC strains tested. Notably, IAA was without effect in all EHEC and EAEC strains tested (Fig. 6c). Together, these data indicate that indole and related derivatives affect a broad range of physiological properties that contribute to virulence of EPEC, EHEC, and EAEC.

### ICA and Indole also Inhibit Virulence in *tnaA*-deficient Bacteria

Indole and its derivatives are produced by commensal *E. coli* strains (Fig. S2c), and inhibit virulence, biofilm formation, and cytotoxicity in EPEC, EHEC, and EAEC strains (Figs. 5,6). These observations led us to consider the possibility that these molecules might also have activity on bacteria that lack tryptophanase and do not produce indole. We tested this possibility on the A/E pathogen *C. rodentium,* which contains the LEE but lacks *tnaA*.

**Figure 7:**
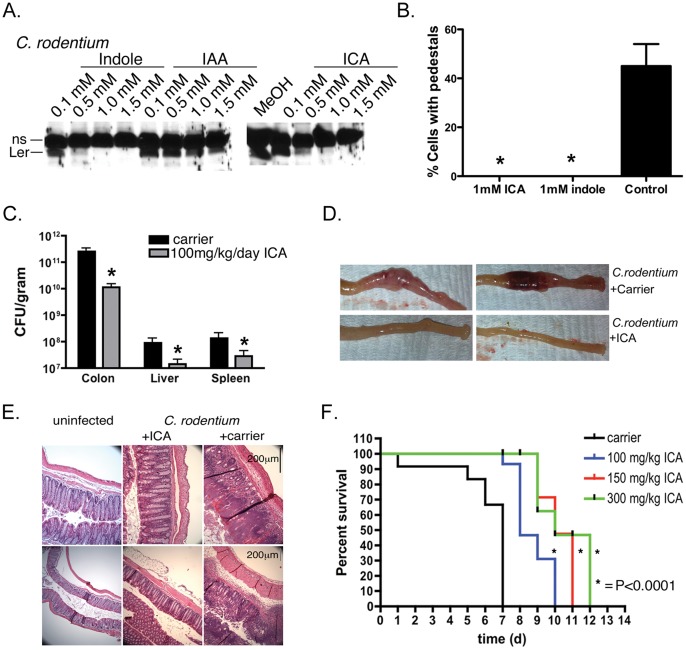
ICA reduces virulence of *C. rodentium in vitro* and *in vivo*. (**a**) Western analysis of Ler expression in *C. rodentium* treated with various concentrations of indole, ICA or IAA. The non-specific band recognized by the Ler pAb served as a loading control. (**b**) Quantitation of pedestal formation in 3T3 cells infected with *C. rodentium* in the presence of indole or ICA at various concentrations. Mean +/− SEM are shown; * corresponds to p<0.01 with respect to untreated control. (**c**) CFU from colon, liver, and spleen of MyD88^−/−^ mice infected for 7 days with *C. rodentium* and administered carrier (DMSO/5%Citric acid/PEG400, (30∶35:35%)) or ICA (100 mg/kg/day) by oral gavage. Mean +/− SEM are shown; * corresponds to p<0.01 with respect to untreated control. 8 mice were used for the control and 9 for the treatment with 100 mg/kg ICA. (**d**) Images of colons from animals left uninfected and treated with ICA (100 mg/kg/day) for 7 days, or infected with *C. rodentium* together with carrier or ICA. (**e**) H&E staining of colon sections from mice left uninfected, or infected with *C. rodentium* for 7d and administered either ICA (100 mg/kg/day) or carrier. (**f**) Survival curves of animals treated with carrier or 100 to 300 mg/kg/day ICA. Each curve is from a representative experiment (n = 3 animals per condition), and each experiment was repeated 3 times. Statistical significance was determined by the Kaplan Meyer test.

Indole and ICA and to a lesser extent IAA inhibited Ler and EspA expression (Fig. 7a; Fig. S4c). An effect on Tir could not be assessed because the EPEC Tir antibody did not cross react with *C. rodentium* Tir. As with EPEC and EHEC, indole and ICA inhibited pedestal formation induced by *C. rodentium* (Fig. 7b). Although the effect of ICA was more potent compared to EPEC or EHEC, these data nevertheless suggest that the regulatory mechanisms modulated by these molecules are conserved amongst A/E pathogens, and that these molecules can affect virulence in bacteria that do not produce them.

### ICA Protects Against A/E Pathogen Infections *in vivo*

We next assessed whether indole or its derivatives affected morbidity and mortality of A/E pathogens *in vivo*. We tested this possibility in MyD88*^−/−^* mice, which unlike wild type animals, succumb to infection with *C. rodentium* [30]. As shown in Fig. 7c, ICA delivered orally at 100 mg/kg/day reduced CFU in the colon by 50 fold, and reduced dissemination of the bacteria to other organs including spleen and liver, where CFUs were also reduced by ∼10 fold (Fig. 7c). Moreover, histological examination of animals treated with ICA showed no indication of isolated intramural colonic bleeding, colonic distension, or hemorrhagic colitis, effects evident in all untreated or carrier-treated animals (Fig. 7d; see also [30]). Likewise, H&E staining of colon sections from carrier and untreated animals infected with *C. rodentium*, but not ICA-treated animals, revealed gangrenous mucosal necrosis characterized by visible bacterial colonies, neutrophil infiltration, mucosal injury, edema, apoptosis, intramural bleeding, and epithelial injury (Fig. 7e; see also [30]). Finally, administration of ICA reduced mortality rates in a dose dependent manner. Thus, 100% of animals treated with ICA survived without detectable signs of disease at a time when all the control animals had succumbed (7d post infection; Fig. 7f). Furthermore, the probability of dying was reduced by 1.7 fold. These data were statistically significant using a Kaplan Meyer test (p<0.0001). Collectively, these data suggest that ICA reduces morbidity and mortality of A/E pathogens *in vivo.*

## Discussion

### Identifying Novel Bacterial Intercellular Signaling Molecules Using *C. elegans* and Metabolomics

*C. elegans* genetics have been used to determine nematode genes that confer host susceptibility or immunity to various pathogens [9]. Notably, bacterial virulence and host susceptibility mechanisms identified in *C. elegans* are often highly conserved. Thus, many bacterial mutants that are avirulent in *C. elegans,* are also avirulent in plants and mammals [9]. Moreover, the same innate immune genes that are deployed against a variety of pathogens in *C. elegans*, are likewise important components of mammalian immune pathways [37].

Using *C. elegans* as a bioassay system in conjunction with NMR-based metabolomics, we have identified a family of bioactive small molecules related to indole, that are produced by pathogenic as well as avirulent commensal *E. coli* strains. These molecules, when presented together, reconstitute the toxicity apparent when *C. elegans* is incubated with EPEC. Moreover, brief exposure of *C. elegans* to these molecules induces a long lasting protective response (Fig. 4). Thus, indoles join a growing class of small molecules produced by bacteria that act as toxins or immunoregulators in *C. elegans*. Other examples include quinolone signal (PQS) and pyocyanin in *Pseudomonas aeruginosa* [38], [39], [40], [41].

### Effects of Toxins in *C. elegans* and Mammals

Several studies have utilized *C. elegans* as a bioassay to identify small signaling molecules from bacteria. For example, phenazines are required for *C. elegans* killing, and are also required for pathogenesis in plants and mice (reviewed in [9]). Other *Pseudomonas* molecules such as pyocyanins are without toxic effects in *C. elegans*, but induce virulence factor expression in the bacteria and inflammatory responses in mammals, both of which contribute to pathogenicity.

We identified indoles based on their toxicity in *C. elegans*. However, unlike pyocyanins, indoles are protective in mammals likely because they suppress expression of bacterial virulence genes and shigatoxin, at least at high concentrations. Moreover, our findings suggest that whereas indole is itself is toxic to C. elegans, indole-like molecules have no evident toxicity in *C. elegans* nor in mice (Fig. S3b; Figure 7f). Our results highlight the utility of *C. elegans-*pathogen systems in conjunction with metabolomics for identifying novel signaling molecules. Indeed, these methodologies may be broadly applicable to identifying small signaling molecules in many organisms.

Whereas we describe here the identification and function of indoles as intercellular signals in *E. coli*, current efforts are underway to identify the bacterial receptors for these molecules. Moreover, indoles may function as pseudosubstrate inhibitors or modulators of pathways in *C. elegans* as well as mammals, and we are using NMR-based methodologies to define how indoles alter normal host metabolomic profiles. In summary, our data highlight the use of *C. elegans* for identification of novel small molecules produced by bacteria. The model may also prove useful as a low-cost high-throughput screening model for identification of derivatives with enhanced potency.

### Indoles as Signaling Molecules in Bacteria

Indole has been identified as a stationary-phase quorum sensing and intercellular signaling molecule in nonpathogenic strains of *E. coli* (Wang et al. 2001), particularly with respect to tolerance to stressors [42], [43], [44]. Thus, secreted indole induces multi-drug transporter genes in *E. coli* [43] as well as other bacteria [45]. Recent reports indicate that drug resistant *E. coli* can, under antibiotic selection, enhance the survival capacity of the overall population by secreting indole, which induces efflux pumps in vulnerable cells, and thereby engenders resistance. Importantly, although such “altruism” depends upon *tnaA* and indole [16], data presented here suggest that a family several different indole derivatives may in fact orchestrate such effects.

A variety of other bacteria also use indole derivatives for signaling functions. Soil bacteria such as *Pseudomonas* and *Erwinia*, lack tryptophanase but synthesize IAA from tryptophan using either the indole-3-acetamide pathway or the indole-3-pyruvate pathway [46]. The indole metabolite 7-hydroxyindole reduces pathogenicity of *Pseudomonas aeruginosa* [47]. Moreover, in accordance with our findings in pathogenic *E. coli,* IAA also suppresses genes encoding the Type III Secretion System and various translocated effectors in *Pseudomonas* [46]. Our data indicate that EPEC produce several indole species at high concentrations in a manner that depends on tryptophanase, though additional low abundance species are evident. Together, these observations suggest that the family of indoles is produced by diverse pathways and have a myriad of functions as intercellular signals in a broad range of bacteria.

### Indoles in Plants and Animals

The effects of indole and its derivatives are not restricted to bacteria, and extend across all domains of life. For example, in mammals, indole derivatives and tryptophans are converted to kynurenine by indoleamine-2,3-dioxygenase (IDO), which is induced by interferon-γ [48], and has been implicated in immune tolerance [49]. Likewise, IAA and related compounds, collectively known as auxins, serve as phytohormones in plants that mediate cell growth and bending responses of coleoptiles towards light [50]. Notably, IAA also regulates virulence in phytopathogens and acts as an intercellular signaling molecule in other soil bacteria [51]. Thus, the co-evolution of plants and bacteria may have facilitated the coordinate use of secreted hormones by both symbiotic and pathogenic strains as well as by the host. Such interkingdom interactions have been reported with adrenergic agonists [52], which regulate both mammalian and bacterial signaling, and we speculate that the indole family may act similarly.

### Indoles Regulate Virulence in Pathogenic *E. coli*

Our data indicate that indoles regulate several aspects of bacterial function associated with pathogenicity in EPEC, EHEC and EAEC. At low concentrations, indole activates expression of the LEE operon in pathogenic *E. coli* (Fig. S4a) [10], [35]. Results presented here suggest that the effects of indole and its derivatives on the LEE depends upon concentration, with µM levels activating transcription, and mM levels suppressing it ([10] and Figs. 5, 7). As a consequence of suppressing Ler in EPEC, expression of genes encoding the Type III Secretion System and various virulence factors such as Tir are reduced, resulting in inhibition of pedestal formation (Fig. 5). Similar effects on virulence and pedestals are seen with EHEC, though whether the effect occurs through Ler is uncertain, as Ler levels are nearly undetectable in EHEC (see also [35]). Effects of indoles extend to other physiological properties associated with virulence as well, including biofilm formation, motility, and perhaps most significantly, Shiga toxin production (Fig. 6). The effects of indoles are conserved in EPEC and EHEC, as well as EAEC, including in strains derived from a recent outbreak in Germany. Moreover, inhibition of virulence extends to *C. rodentium*, which does not itself produce indoles, in accordance with the idea that transcriptional regulation by this class of molecules is not only conserved amongst pathogenic *E. coli*, but perhaps more broadly as well.

### Why do *E.coli* Produce Indoles?

Both commensal and pathogenic strains of *E. coli* produce indole and ICA, and mM concentrations of indole have been detected in feces [53], and we are currently evaluating the concentration of indole derivatives in human fecal samples. Our observations raise the question as to the function of indoles in the intestinal tract. For an intestinal pathogen such as EPEC or EHEC, an indole-based detection mechanism may facilitate competition for limited resources with commensal strains during early stages of infection. During these stages, the pathogen may outcompete commensal strains by suppressing virulence mechanisms that are costly to fitness, in favor of upregulating other pathways critical for survival. Our preliminary data suggest that whereas indole and indole derivatives affect some pathways associated with stress responses (e.g. biofilm, motility etc.), not all stress pathways are similarly affected (B. B. and D. K. unpublished).

Alternatively, indoles produced by commensal *E. coli* may serve as anti-virulence factors that neutralize intestinal pathogens. Alteration in commensal flora resulting in reduced production of indole derivatives may increase susceptibility to infection. Conversely, indoles such as IAA are prevalent in plants and may influence both commensal and pathogenic bacteria upon digestion. As such, indoles obtained from dietary sources may play a protective role. Such mechanisms might in part explain the variable penetrance of disease in individuals exposed to the EHEC, a prospect we are currently testing.

### Indoles as Therapeutic Agents

Our data showing decreased virulence and reduced mortality upon oral administration of ICA in MyD88^−/−^ mice infected with *C. rodentium* raises the possibility that administration of exogenous indoles may represent a novel means to prevent or mollify infections caused by pathogenic *E. coli*. Moreover, our observation that these molecules also reduce expression of STX in EHEC strains suggest a means to ameliorate STX-mediated pathology following infection, a key contributor to morbidity and mortality in EHEC and EAEC. Notably, because indoles do not affect bacterial growth *per se*, they may not easily engender resistance compared to conventional antibiotics, and they may prove effective against strains resistant to conventional antibiotics. Finally, in contrast to antibiotics, these compounds may not adversely perturb populations of commensal bacteria.

Development of therapeutic agents for pathogenic *E. coli* has traditionally targeted individual components of the virulence machinery such as the type III secretion systems, or effector molecules, an approach that has not proven particularly effective (Rasko and Sperandio, 2010). ICA represents a potentially unique therapeutic agent as it affects multiple virulence determinants including the type III secretion system, effector molecule production, biofilm formation and STX production in both LEE and non-LEE *E. coli* strains (Figs. 5 and 6). There is a strong mandate to develop therapeutic agents for pathogenic *E. coli* infections, and particularly those that broadly affect virulence. For EHEC and EAEC, antibiotics are contraindicated because they are thought to lyse bacteria thereby releasing more STX and exacerbating morbidity and mortality. Moreover, no antibiotic or small molecule identified to date affects STX production or levels.

Our preliminary structure-function analysis identified molecular features on the indole scaffold required for inhibition of virulence (e.g. the carboxaldehyde at position 2 or 3), as well as chemical groups that provide additional or improved functionality *vis a vis* clinically relevant properties. For example, a nitro group at position 4 (**5**) results in suppression of bacterial growth, and, independently, enhancement by 10 fold of anti-shigatoxin activity. In general, more polar substituents such as the nitro (**5**), carboxynitrile (**6**) and carboxylic acid-methyl ester (**7**) groups imparted additional benefit to the carboxaldehyde series, whereas less polar substituents did the opposite. Mechanistically, additions to the indole ring may alter the electrophilicity of the carboxaldehyde at position 3. Alternatively, such modifications may change interactions of the molecule with its target(s), or provide access to new targets. These data will not only facilitate exploration of the mechanism by which indoles act, but also will serve to guide for further drug development of this series of compounds.

In summary, by using *C. elegans* in combination with NMR- and MS-based comparative metabolomics, we have obtained data that provide an important advance in the identification of small molecules that regulate multiple aspects of bacterial physiology and pathogenicity. In particular, we identified a family of indoles that regulate virulence *in vitro* and *in vivo*. Notably, some of the indoles produced by commensal *E. coli* and are also present in dietary sources. Thus, these molecules may represent components of the natural host defense against infection. Finally, when delivered exogenously, indoles may represent a new class of “signaling molecule-based” therapeutic agents for treating infections caused by pathogenic *E. coli*.

## Supporting Information

Figure S1 Structures of natural and commercially available synthetic indole derivatives used for structure-activity profile.
(TIF)

Figure S2 Identification of indole as an *E. coli* toxin. **(a)** Survival curves of *C. elegans* N2 and various mutants upon exposure to 3.5 mM indole in agar plates. Similar results were obtained upon exposure of *C. elegans* in broth. No adjustment for strain differences was made to these data. **(b)** *E. coli* strains kill *C. elegans*. *C. elegans* N2 animals were exposed to *E. coli* P90C, MG1655 or OP50 grown on LBT plates for 6 hours, and then transferred to NGM agar containing OP50 for 24 hours and survival assessed. **(c)** *E. coli* and EPEC strains secrete different amounts of indole or its derivatives. **(d,e)** Pre-exposure of *C. elegans* N2 (**d**) or various mutants (**e**) to 3.5 mM indole in LB agar followed by a subsequent challenge with 3.5 mM indole in LB agar. Only N2 is conditionable. For d,e, mean +/−95% confidence intervals are shown. Lack of overlapping error bars indicates significance at the 5% level.
(TIF)

Figure S3 **(a)** Overlay of UV-HPLC chromatograms (absorption at 260 nm) of the EPEC and EPECΔ*tnaA* extracts as well as of synthetic standards of ICOOH, ICA, IAA, and indole, obtained using a reverse phase HPLC column. Effects of indole derivatives on *C. elegans* and on infection of mammalian cells. **(b)** Neither ICA, nor IAA, nor ICOOH alone at the indicated concentrations nor in combination killed *C. elegans*. **(c)** Pre-exposure to ICA or indole increased survival slightly upon subsequent exposure to EPEC, whereas IAA was less effective.
(TIF)

Figure S4 **(a)** Western analysis of Tir expression in EPEC treated with low concentrations of indole, ICA or IAA. Note that indole and ICA induce Tir expression at low concentrations, but all three compounds (indole, ICA and IAA) suppress Ler and Tir at high concentrations. The band recognized by the DnaK pAb served as a loading control. **(b)** Growth curves of EPEC, EHEC O157:H7 strain EDL933, or *C. rodentium* in the presence of various concentrations of indole, ICA, or the carrier methanol. The synthetic indole derivative 4-Nitroindole-3-carboxaldehyde did however suppress growth of EPEC, *C. rodentium*, and EAEC 3493. **(c)** Western analysis of secreted EspA from *C. rodentium* or EHEC treated with various concentrations of ICA.
(TIF)
