# Supplementary figures and images for: A Family of Indoles Regulate Virulence and Shiga Toxin Production in Pathogenic E. coli
Source: PLoS One. 2013 Jan 23;8(1):e54456. doi: 10.1371/journal.pone.0054456 (PMC3553163; doi:10.1371/journal.pone.0054456)

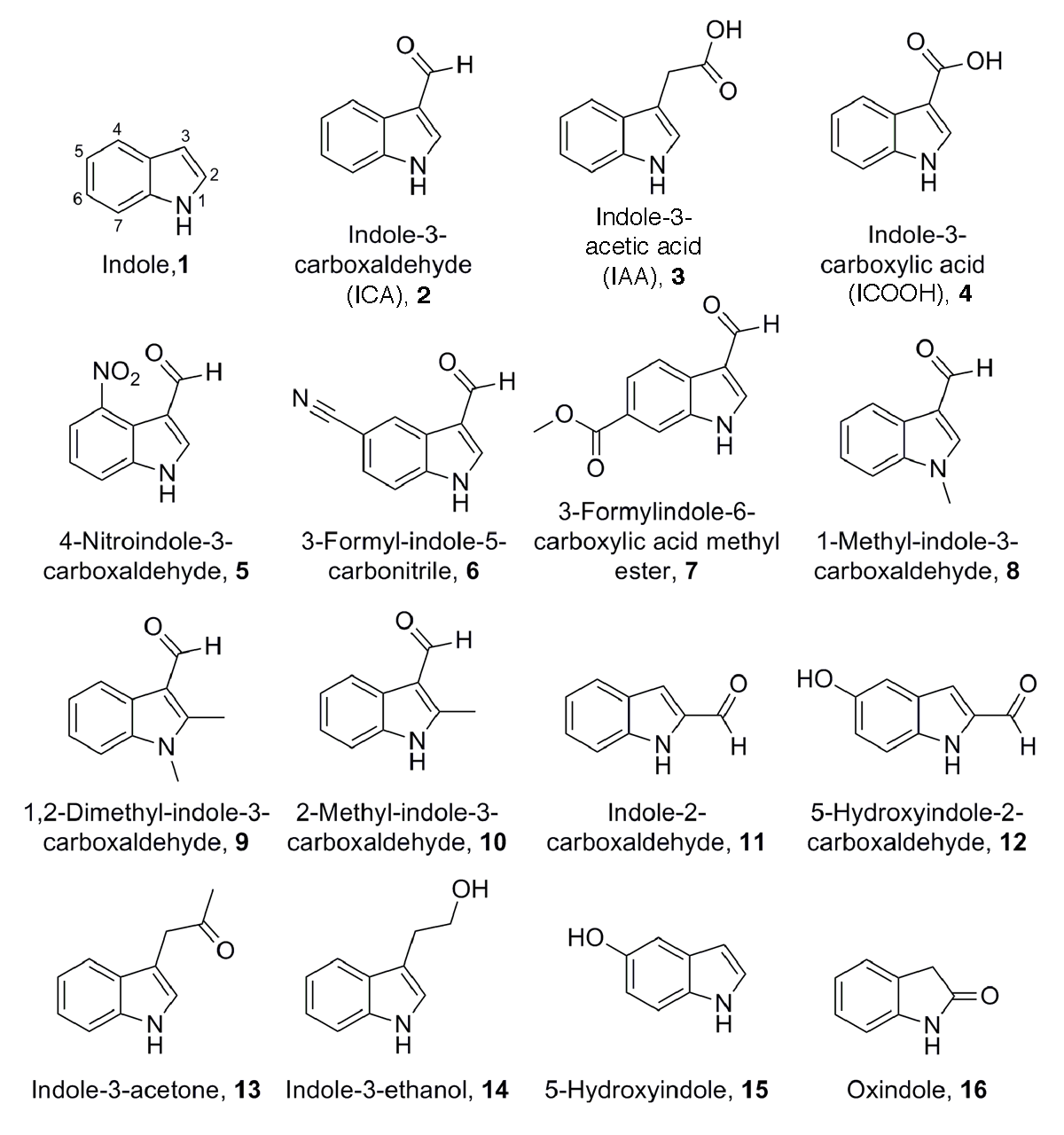

Supplement: Figure S1 — Structures of natural and commercially available synthetic indole derivatives used for structure-activity profile. (TIF) [file pone.0054456.s001.tif]

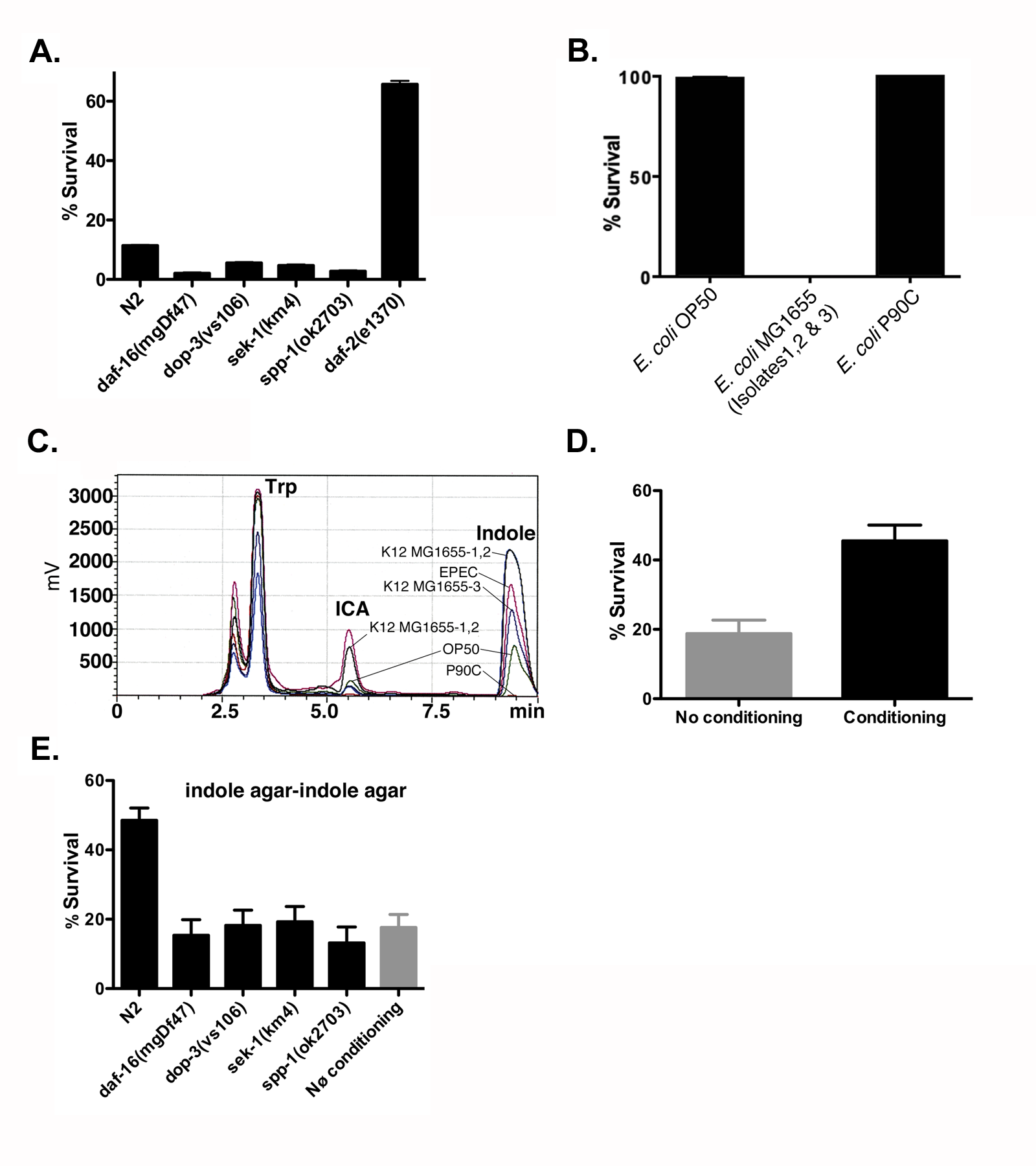

Supplement: Figure S2 — Identification of indole as an E. coli toxin. (a) Survival curves of C. elegans N2 and various mutants upon exposure to 3.5 mM indole in agar plates. Similar results were obtained upon exposure of C. elegans in broth. No adjustment for strain differences was made to these data. (b) E. coli strains kill C. elegans. C. elegans N2 animals were exposed to E. coli P90C, MG1655 or OP50 grown on LBT plates for 6 hours, and then transferred to NGM agar containing OP50 for 24 hours and survival assessed. (c) E. coli and EPEC strains secrete different amounts of indole or its derivatives. (d,e) Pre-exposure of C. elegans N2 (d) or various mutants (e) to 3.5 mM indole in LB agar followed by a subsequent challenge with 3.5 mM indole in LB agar. Only N2 is conditionable. For d,e, mean +/−95% confidence intervals are shown. Lack of overlapping error bars indicates significance at the 5% level. (TIF) [file pone.0054456.s002.tif]

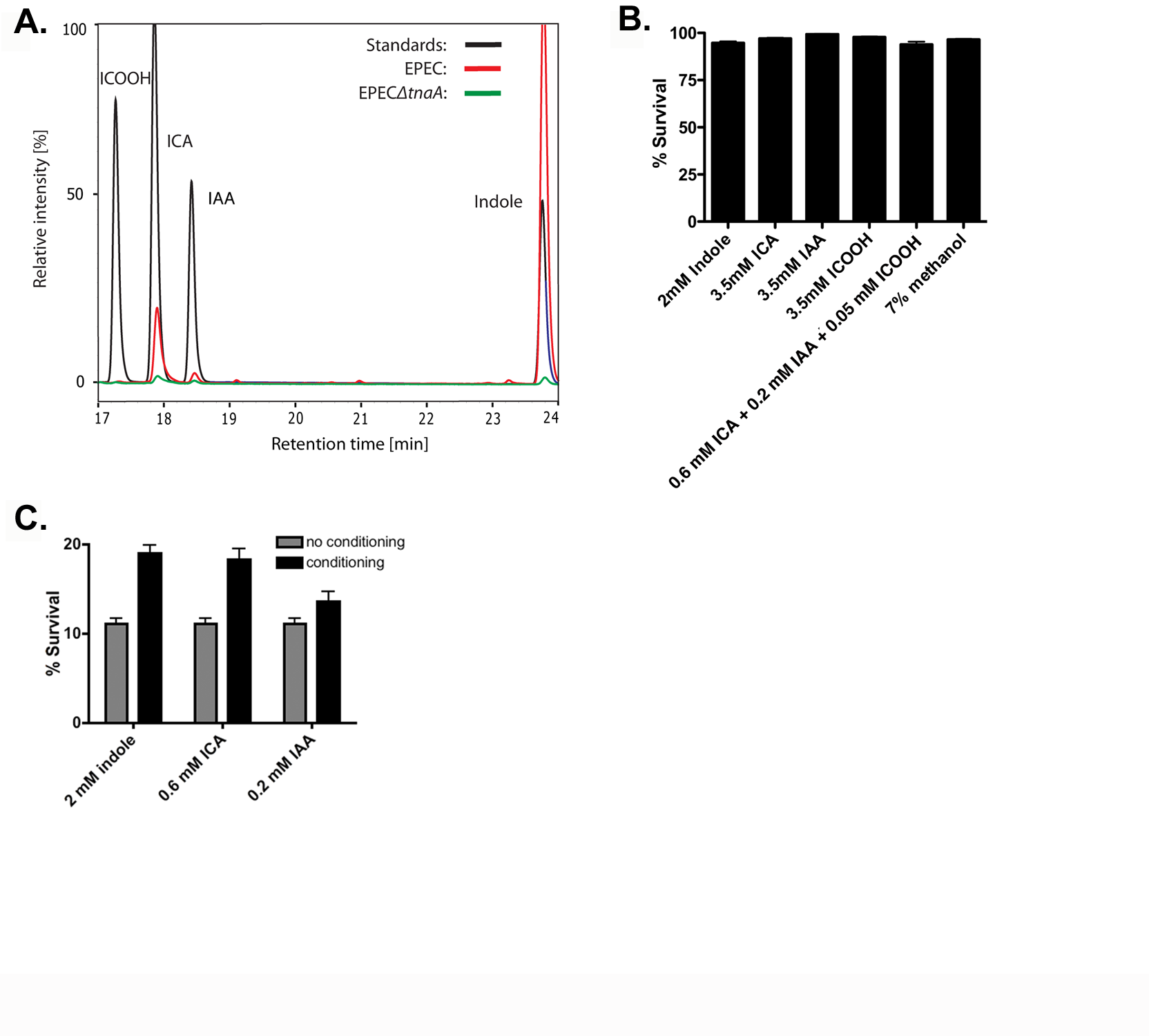

Supplement: Figure S3 — (a) Overlay of UV-HPLC chromatograms (absorption at 260 nm) of the EPEC and EPECΔtnaA extracts as well as of synthetic standards of ICOOH, ICA, IAA, and indole, obtained using a reverse phase HPLC column. Effects of indole derivatives on C. elegans and on infection of mammalian cells. (b) Neither ICA, nor IAA, nor ICOOH alone at the indicated concentrations nor in combination killed C. elegans. (c) Pre-exposure to ICA or indole increased survival slightly upon subsequent exposure to EPEC, whereas IAA was less effective. (TIF) [file pone.0054456.s003.tif]

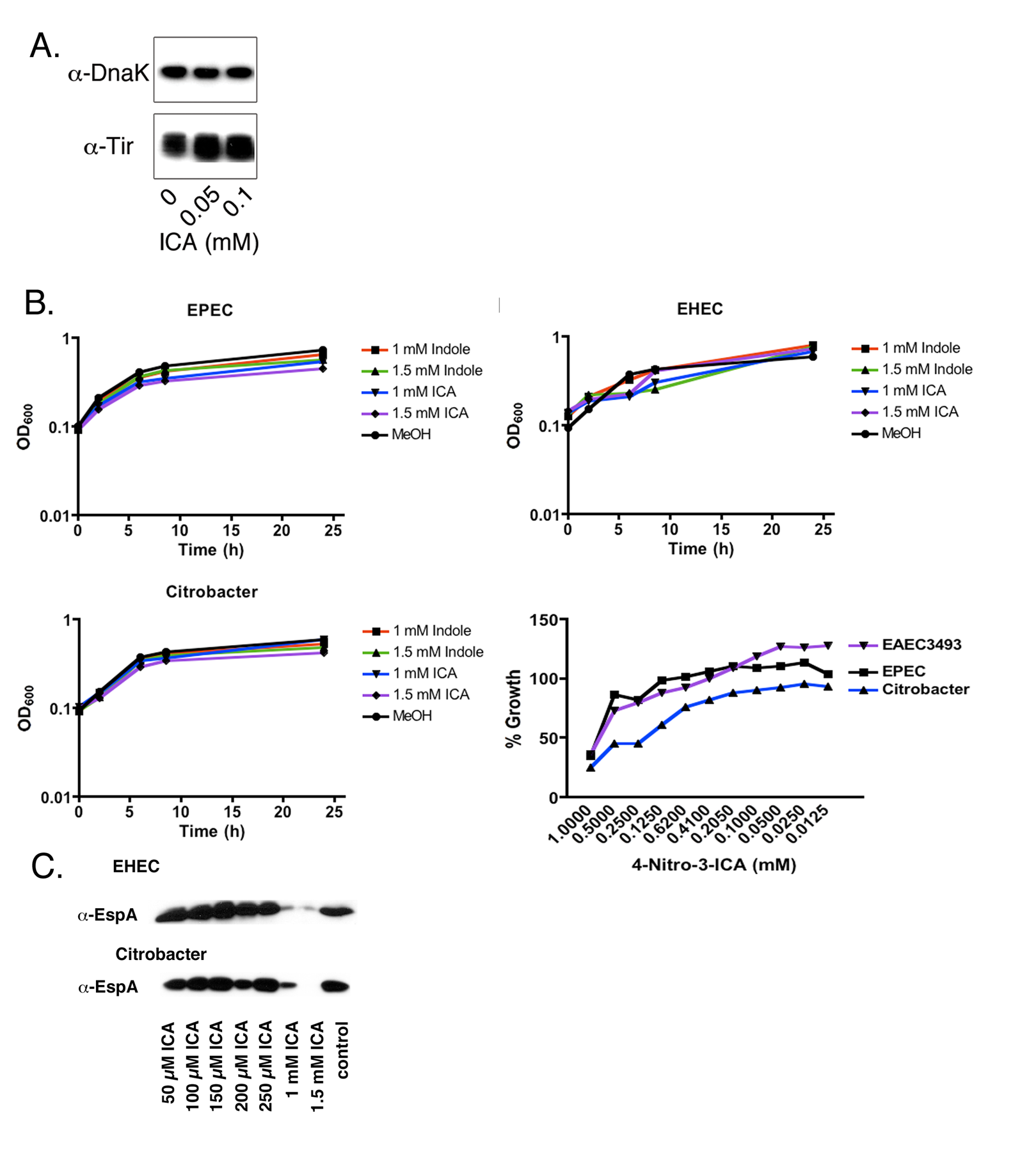

Supplement: Figure S4 — (a) Western analysis of Tir expression in EPEC treated with low concentrations of indole, ICA or IAA. Note that indole and ICA induce Tir expression at low concentrations, but all three compounds (indole, ICA and IAA) suppress Ler and Tir at high concentrations. The band recognized by the DnaK pAb served as a loading control. (b) Growth curves of EPEC, EHEC O157:H7 strain EDL933, or C. rodentium in the presence of various concentrations of indole, ICA, or the carrier methanol. The synthetic indole derivative 4-Nitroindole-3-carboxaldehyde did however suppress growth of EPEC, C. rodentium, and EAEC 3493. (c) Western analysis of secreted EspA from C. rodentium or EHEC treated with various concentrations of ICA. (TIF) [file pone.0054456.s004.tif]
